# A Handbook of Medical Climatology

**Published:** 1899-06

**Authors:** 


**A Handbook of Medical Climatology.** By S. Edwin Solly, M.D. Pp. 470. Philadelphia: Lea Brothers & Co. 1897.

This book is intended for the use of three classes of persons: "the present generation of physicians who prescribe travel, the travelers themselves, and the rising generation of medical students should be taught to recognize the principles of medical climatology and its proper relation to general therapeutics." The author rightly summarises much of the mass of literature on the subject as a "desert of rubbish," in which commonly each writer claims for his own resort "the ability to cure all diseases, and the only invalids warned against coming are those in whom disease is far advanced." We may fairly class this book as one of the "bright oases of truth and reason" to which the author alludes. There are three sections: the first deals broadly with general principles of medical climatology, the second treats of climate in relation to disease, and the third is devoted to special climates in selected resorts.

The question has been asked,[1] "What constitutes a good climate for the healthy?" The reply is, that one with constant moderate variations calls forth the energy of the different organs and functions, their power of adaptation and resistance, and keeps them in a working condition. Such are the climates of England all the year round: and they belong to the most health-giving in the world; for here we see the finest trees, the finest animals, the finest men—and they are most conducive to longevity, although not always the most agreeable or exhilarating. The climates for invalids are those with an abundant supply of pure air and water, the possibility of spending a great part of the day in the open air, good hygienic and dietetic arrangements, and the presence of a good local physician. Our own country—"Le pays ou il fait froid toujours, ou le soleil resemble à la lune et la lune à un fromage à la crême" (Dumas)—is not, however, the one where Dr. Solly derives his principal experience; and we naturally associate his name with the treatment of phthisis at the elevated health-resorts of Colorado.

As to the nature of the influence of altitude, he shows that the diminished barometric pressure is the principal factor; and after giving interesting information on the hæmatogenic effects of high altitude, more especially in consumptives, he quotes the experiments of Egger, made at Arosa in 1891, showing a marked increase of 16 per cent. in the number of the red blood cells. From an analysis of eight thousand cases he deduces the statement that there is a steady rise in the percentage of improvement as we go upwards: "That the majority of consumptives do better, other things being equal, the further they are removed from the sea, and that they do better in high than in low altitudes, wherever situated" (p. 137). "Le fait est donc pour nous indubitable, on ne devient pas phthisique à Quito" (p. 101). Gardiner's researches on the immunity found at high altitudes were commented on by us in the June number of 1898 (p. 127). We look upon the seventy-three pages on phthisis and climate as the most valuable part of the work. Dr. Solly has had a large experience and can speak with authority. His *résumé* of the indications and contra-indications in the climatic treatment of phthisis should be read by all on whom the responsibility rests of choosing a health-resort for those patients. Section III. gives an excellent outline of the different climates of all parts of the globe.

---

**The Mineral Waters and Health Resorts of Europe.** By HERMANN WEBER, M.D., and F. PARKES WEBER, M.D. Pp. xiii., 524. London: Smith, Elder, & Co. 1898.

Known in its original issue as *The Spas and Mineral Waters of Europe*, this classical work has undergone much alteration and

[1] Clifford Allbutt's *System of Medicine*, 1896, vol. i. p. 284.

